# Natural genetic variation and hybridization in plants

**DOI:** 10.1093/jxb/erx377

**Published:** 2017-11-17

**Authors:** Ian R Henderson, David E Salt

**Affiliations:** 1 Department of Plant Sciences, Downing Street, University of Cambridge, Cambridge, CB2 3EA, United Kingdom; 2 Division of Plant and Crop Sciences, School of Biosciences, University of Nottingham, Sutton Bonington Campus, Sutton Bonington, LE12 5RD, United Kingdom

**Keywords:** Agriculture, DNA sequencing technologies, epistasis, evolutionary adaptation, genetic variation, intra- and inter-specific hybridization, meiotic recombination, phenotypic traits, reproductive strategy (flowering time)

**Extensive genetic variation underpinning phenotypic traits exists in natural plant populations, and this is intimately connected to the process of evolutionary adaptation. Importantly, such genetic variation also exists in the close relatives and progenitor species of many of our staple crops, which has great potential significance in agriculture. Therefore, the study of plant natural genetic variation can provide new insights into biological mechanisms, in addition to providing a vital source of genetic diversity in crop breeding.**

Humans have a long history of exploiting natural variation via hybridization in plants, for example during domestication of crops from wild species (Doebley *et al.*, 2006). An example is the domestication of modern bread wheat via two successive inter-species hybridizations, resulting in a complex hexaploid genome (Mayer *et al.*, 2014). Today, understanding how natural genetic polymorphisms (i.e. genotype) translate into phenotype is a central question, with relevance to how plant populations adapt to changing environments. This is particularly relevant with respect to climate change and understanding how crops will adapt to the resulting alterations in temperature and water availability. To address these and related questions the 1001 Genomes Project has provided high resolution insight into global genetic and epigenetic polymorphisms in the model plant *Arabidopsis thaliana* (Arabidopsis) (Box 1; Alonso-Blanco *et al.*, 2016; Kawakatsu *et al.*, 2016). The resources generated from this project will facilitate further understanding of the molecular basis of adaptation in this species, with relevance more broadly to other plants and animals. For example, it has emerged that DNA methylation shows a clear latitudinal pattern in Arabidopsis, associated with variation in *trans* modifiers (e.g. DNA methyltransferases) (Dubin *et al.*, 2015; Kawakatsu *et al.*, 2016).

**Box 1. The 1001 Genomes Project**

As more whole-genome seque nces were completed for various wild-collected accessions of Arabidopsis it became clear that the concept of the single species *Arabidopsis thaliana* genome was problematic. Extensive polymorphisms exist between different *A. thaliana* individuals collected from different locations, and genes thought to be non-functional from the first *A. thaliana* genome were found to be functional in other individuals. Such considerations gave rise to the 1001 genomes project (1001genomes.org). Sequencing over 1000 individuals collected from different geographic locations would allow a fuller characterization of the genetic variation within the species, providing a powerful resource for further investigations.

The image shows an educational installation within the Cruickshank Botanic Gardens (University of Aberdeen) focused on genomics called ‘a living-plant double helix’ created by Liam Salt from the diverse set of over 1000 different *A. thaliana* accessions used in the 1001 genomes project.

**Figure:**
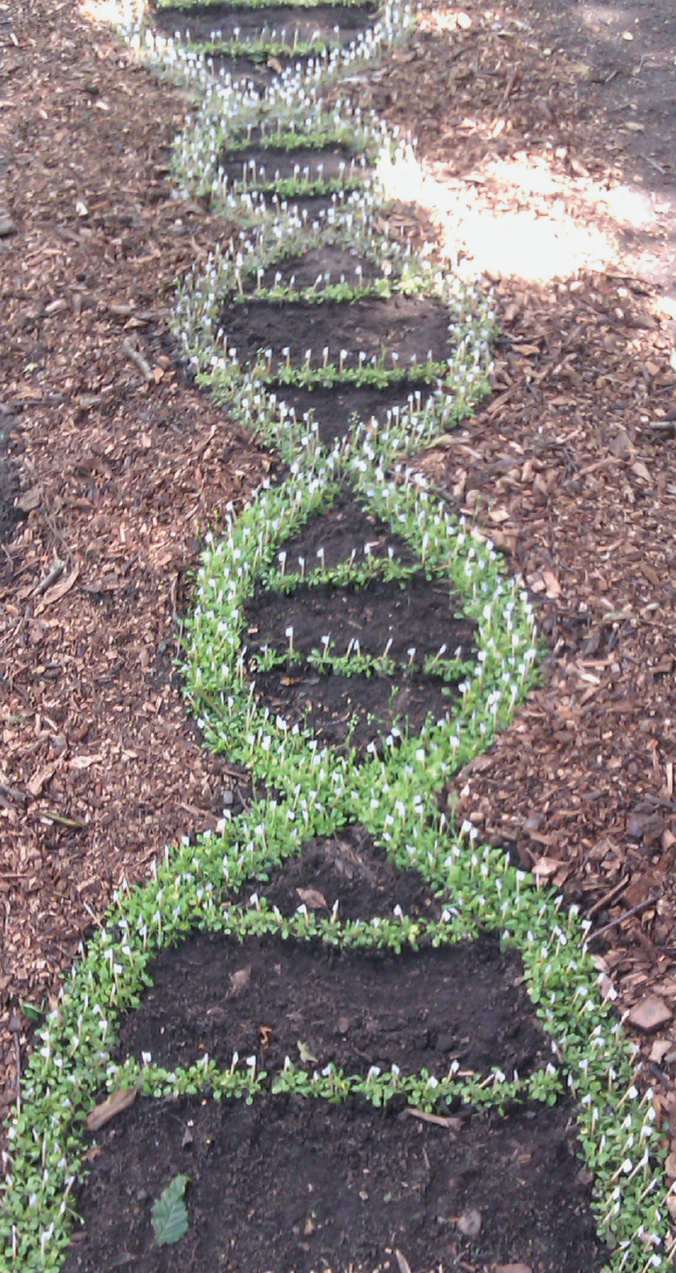

These and similar projects in other plant species, including many crop species, are being greatly strengthened by new DNA sequencing technologies that allow unprecedented depths of sequencing and longer reads. This is particularly valuable when repetitive elements of the genome, such as transposons and centromeres, are of interest (Quadrana *et al.*, 2016; Stuart *et al.*, 2016;). For example, Legget and Clark review the application of nanopore technology to genome sequencing (Legget and Clark, 2017). The compact size of nanopore technology opens the possibility of performing hitherto challenging sequencing experiments in the field, and its low cost has the potential to accelerate genomics.

## Genotype, phenotype and epistasis

In order for natural genetic variation to be utilized in agriculture, backgrounds of interest are typically crossed to domesticated strains, via either intra- or inter-specific hybridization. However, the phenotypic expression of traits can be complex, and the inheritance of characteristics of interest may not be easy to predict. Hence, the interaction observed between genotypes at different loci that determine phenotype has emerged as an area of great interest. Forsberg and Carlborg (2017) discuss examples of complex interactions between variants during phenotypic expression, using both theoretical and experimental observations. They provide detailed analysis of the roles of epistasis and genetic variance heterogeneity in phenotypic expression. These concepts are examined as examples of non-additive genetic inheritance patterns that probably contribute to the variation observed in plants (Forsberg and Carlborg, 2017). Understanding such patterns of genetic interaction will be important to fully utilize genetic variation during crop improvement.

## Flowering, hybrids and meiosis

Quantitative trait loci (QTL) analysis is a powerful approach to understand complex multigenic traits in plants and animals. Indeed, QTL mapping has been a successful approach for dissecting the molecular basis of flowering-time control in Arabidopsis, as reviewed by Bloomer and Dean (2017). These approaches have identified two major genes, FLOWERING LOCUS C (FLC) and FRIGIDA (FRI), that determine Arabidopsis life history; hence, the species may be either a vernalization-requiring winter annual or a rapid cycler (Bloomer and Dean, 2017). Natural variation in these genes underpins evolution of diverse life history strategies within Arabidopsis that has allowed it to colonize a wide geographic range, spanning different environmental conditions (Bloomer and Dean, 2017). Studies in Arabidopsis have identified key genes controlling flowering time and elucidation of their molecular mechanisms, including epigenetic silencing of *FLC* by vernalization, which involves the Polycomb repression system and long non-coding RNAs (Bloomer and Dean, 2017).

Flowering is a key trait that can determine the extent of plant inter- and intra-specific hybridization (Schmickl *et al.*, 2017). Yant and colleagues examine how hybridization can lead to allele introgression and be connected to speciation events (Schmickl *et al.*, 2017). As discussed, the utility of new DNA sequencing technologies is accelerating study of these phenomena (Legget and Clark, 2017). Sequencing of populations occurring at hybrid zones is discussed as an approach for identifying introgressed adaptive alleles, in addition to examples of horizontal gene transfer (HGT) between species. Importantly, application of deep-sequencing technologies is proving to be a powerful approach for identifying genomic islands of divergence associated with adaptive allele introgression. Such islands are typically detected as regions with high genetic divergence compared to non-differentiated regions elsewhere in the genome (Schmickl *et al.*, 2017).

Once hybrids form, natural variation can be further recombined via the meiotic cell division (Hunter, 2015). Meiosis generates haploid gametes from diploid progenitor cells, using a single round of DNA replication followed by two rounds of chromosome segregation (Hunter, 2015). Importantly, during the first meiotic cell division homologous chromosomes pair and undergo recombination, which can result in reciprocal crossover (Hunter, 2015). Henderson and colleagues review how, in addition to reassorting natural variation, sequence polymorphism can itself modify the meiotic recombination process (Lawrence *et al.*, 2017). This can occur by both *cis*- and *trans*-acting effects of variation, and QTL-mapping approaches have been successful in identifying genes controlling recombination, including the conserved E3 ligase gene *HEI10* (Lawrence *et al.*, 2017; Ziolkowski *et al.* 2017). Plants also show high rates of polyploidy, which can create challenges for stable genome transmission during meiosis (Otto, 2007). Importantly, genetic variation and loci associated with stable meiotic inheritance of polyploid genomes have also been identified (Griffiths *et al.*, 2006; Yant *et al.* 2013).

## Future directions

Natural genetic and epigenetic variation in plants is extensive, and remains to be fully described and harnessed in crop species. Nevertheless, new sequencing technologies provide opportunities to explore plant variation at greater depth and accuracy. This is particularly important in species with large, repetitive genomes, or in chromosomal regions typified by repeated sequences. For example, centromeres and ribosomal RNA clusters remain poorly understood due to our inability to completely sequence these regions. As technology advances it will be possible to obtain near-complete descriptions of genetic variation in natural populations. This will provide the opportunity to examine patterns of variation in relation to environmental adaptation at unprecedented resolution in space and time. As our climate continues to change, understanding global patterns of natural genetic variation and its relationship to plant adaptation will thus be of critical importance.
